# Clinical characteristics in patients with hereditary amyloidosis with Glu54Gln transthyretin identified in the Romanian population

**DOI:** 10.1186/s13023-020-1309-9

**Published:** 2020-01-30

**Authors:** Andreea Jercan, Amalia Ene, Ruxandra Jurcut, Mirela Draghici, Sorina Badelita, Mihaela Dragomir, Camelia Dobrea, Monica Popescu, Dumitru Jardan, Emanuel Stoica, Speranta Iacob, Ionela Codita, Claudiu Stan, Daniel Coriu

**Affiliations:** 1 Department of Hematology, Fundeni Clinical Institute, no. 258 Bucharest, Romania; 2 University of Medicine and Pharmacy “Carol Davila”, Bucharest, Romania; 3 Neurology Department, Emergency University Hospital, Bucharest, Romania; 4 Expert Center for Rare Genetic Cardiovascular Diseases, Emergency Institute for Cardiovascular Diseases “Prof. Dr. C.C. Iliescu”, Bucharest, Romania; 5 Emergency Institute of Cardiovascular Diseases “Prof. Dr. C.C. Iliescu”, Bucharest, Romania; 6 Neurology Department, Elias Emergency University Hospital, Bucharest, Romania

**Keywords:** Hereditary amyloidosis, Transthyretin amyloidosis, *Glu54Gln* transthyretin variant, Romania, Cardiomyopathy

## Abstract

**Background:**

In Romania, 23 patients have been diagnosed with hereditary transthyretin amyloidosis (ATTRh), 18 of whom have the *Glu54Gln* mutation. This retrospective cohort included all patients with *Glu54Gln*-mutated ATTRh who were diagnosed in Romania from 2005 to 2018.

**Results:**

Of 18 patients, 10 were symptomatic, five were asymptomatic carriers and three died during the study. All originated from North-East Romania. Median age at symptom onset was 45 years; median age at death was 51 years. All patients had cardiac involvement, including changes in biomarkers (mean N-terminal-pro B-type natriuretic peptide: 2815.6 pg/ml), electrocardiography (15% atrial fibrillation, 38% atrioventricular block, 31% right bundle block), and echocardiography (mean interventricular septum: 16 mm, mean left ventricular ejection fraction: 49%). Scintigraphy showed myocardial radiotracer uptake in all patients. In addition, 92% of patients had polyneuropathy at diagnosis and 53% had carpal tunnel syndrome; 69% exhibited orthostatic hypotension and 31% suffered from diarrhea. No renal or liver involvement was observed.

**Conclusions:**

This is the largest *Glu54Gln*-mutated ATTRh cohort diagnosed to date, and to our knowledge the first describing this variant worldwide. Clinical features of this variant are early onset, neurological and cardiac involvement, aggressive disease progression and short survival. Early diagnosis and therapeutic intervention have potential to improve prognosis in ATTRh.

## Background

Hereditary transthyretin amyloidosis (ATTRh) is a systemic illness characterized by extracellular deposition of amyloid fibrils. These are composed of transthyretin with a modified primary structure caused by point mutations in the transthyretin (TTR) gene. Alteration of the primary structure of TTR induces destabilization and aggregation of fibrils in various tissues, especially in the nervous and cardiac systems, causing severe dysfunction [1]. The age of onset is typically > 30 years and the clinical manifestations depend on which tissue is predominantly affected: ATTRh can manifest itself as peripheral and autonomic neuropathy (neurological phenotype), or as heart-infiltrative cardiomyopathy (cardiac phenotype). Both phenotypes can also occur in the same patient (mixed cardiac/neurological phenotype) [2].

ATTRh is an autosomal dominant inherited illness with variable penetrance [3]. At present, over 120 TTR gene mutations related to ATTRh have been identified [1]. Amongst these gene variants there is considerable heterogeneity in terms of incidence and prevalence, age at symptom onset and clinical manifestation. These factors, together with a low level of awareness of transthyretin amyloidosis (ATTR) among physicians, have led to ATTR being considered a rare illness confined to specific geographical areas. Owing to recent advances in diagnosis and therapy, new geographic areas with an increased incidence of ATTR (such as the Balkans) have been identified, as well as cases in non-endemic areas.

In Romania, the first cases of ATTRh with the *Glu54Gln* mutation were reported in 2012 [4]. So far, 23 patients have been diagnosed with ATTRh in Romania: three with the *Val30Met* mutation, two with the *Glu89Lys* mutation and 18 with *Glu54Gln* mutation. We carried out a retrospective, observational study to describe the clinical characteristics of ATTR in Romanian patients with the *Glu54Gln* mutation.

## Results

Overall, 18 patients were included in the study, of whom 13 had symptomatic disease and five were asymptomatic carriers. At the time of analysis, 10/13 symptomatic patients are still alive and three had died during the study. Asymptomatic carriers were either relatives of diagnosed patients (*n* = 3) or were diagnosed during active screening in the area in which the highest prevalence of ATTR was observed (*n* = 2).

**Fig. 1:**
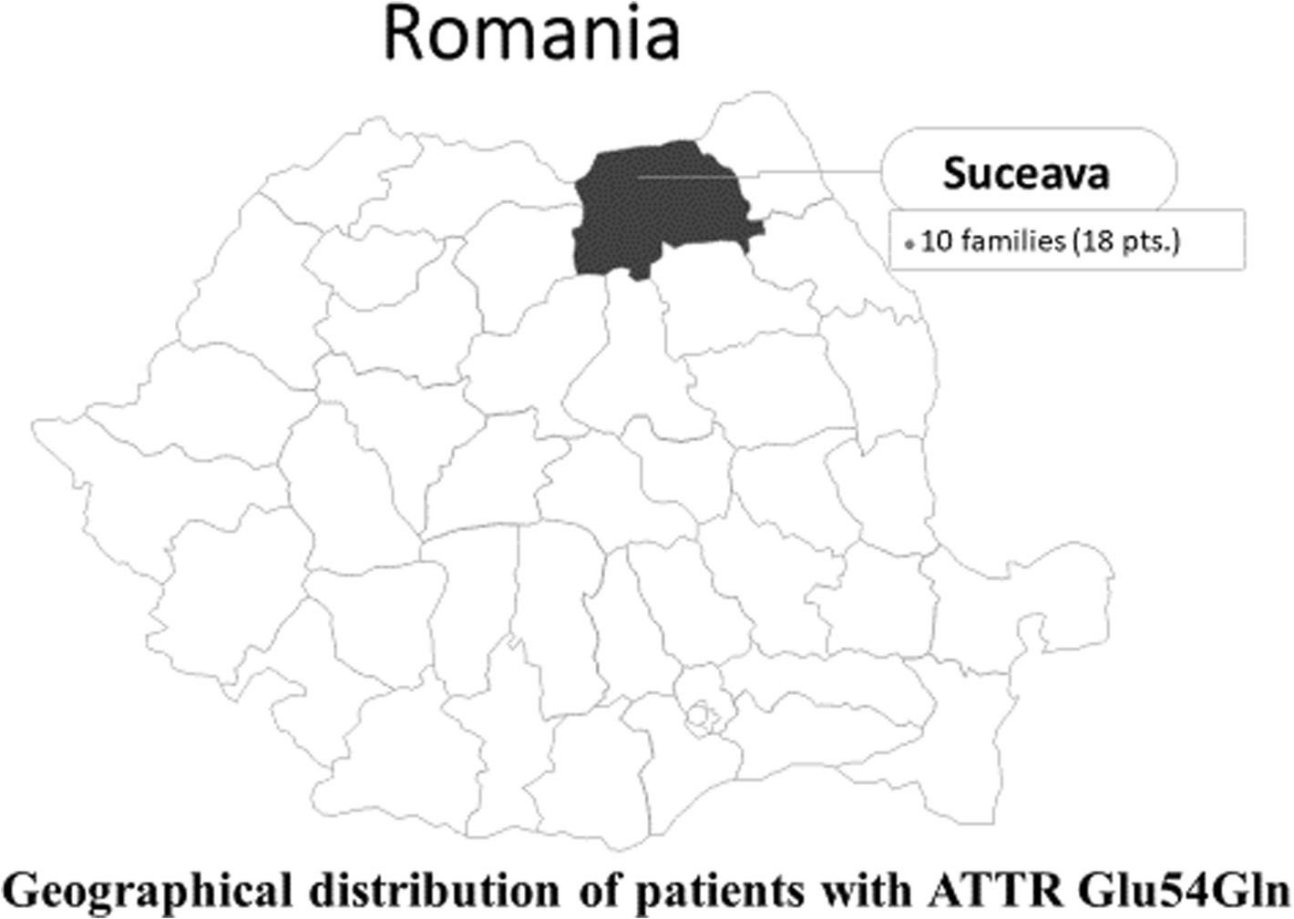
Geographical distribution of patients diagnosed with Glu54Gln-mutated ATTR in Romania. There is a cluster of patients in the North-East area of Romania, in Suceava County. Most patients come from a single town in this county, Todiresti. “Free Romania Editable Map” by yourfreetemplates.com is licensed under CC BY-ND 4.0

All 18 patients come from ten apparently unrelated families originating from North-East Romania (Suceava County); all belong to the Romanian ethnic group (Fig. 1). In nine of these families, the proband was identified as they had symptomatic disease. In the tenth family, the proband was a healthy carrier who was identified by active screening that we carried out in families based in the area. All symptomatic patients had a family history of neurological involvement or death at a young age.

### Prevalence

Prevalence of ATTRh (the number of patients diagnosed with ATTR per 1,000,000 people) was based on the number of diagnosed patients and the current Romanian population according to the National Institute of Statistics. According to these data, Romania has a population of 19,530,631, and Suceava County has a population of 626,789 inhabitants [5]. The prevalence of ATTRh is therefore 1.02 per million, and the prevalence of *Glu54Gln*-mutated ATTR is 0.76 per million. Within Suceava County, the prevalence of *Glu54Gln*-mutated ATTR is 2.39 per 100,000 inhabitants. On 31 December 2018, 20 patients with ATTRh were living in Romania, 15 of whom had the *Glu54Gln* mutation.

### General data on Glu54Gln-mutated ATTR

Of the 18 patients in this study, eight (44%) were male and 10 (56%) were female. Of these, five were asymptomatic carriers (age range: 16–32 years), 10 were symptomatic patients (age range: 35–53 years), and three patients had died during the study (Table 1). Asymptomatic carriers were diagnosed from 2011 onwards and are periodically evaluated.

**Table 1:** Characteristics of symptomatic ATTR patients with the Glu54Gln mutation

*Patient [family]*	*Age at diagnosis*	*Diagnosis (year)*	*Mean time from diagnosis to onset (months)*	*Symptoms at onset*	*Peripheral nervous system dysfunction*	*Autonomic dysfunction*	*Cardiac features*	*Alive/deceased*
*1 [A]*	48	2005	36	CTS Paresthesia	SM-PN CTS	OHT Dysphagia Diarrhea	HF	Deceased
*2 [B]*	43	2008	36	CTS	CTS	–	HF	Deceased
*3 [C*_1_*]	35	2012	11	HF Weight loss Paresthesia	Paresthesia	Diarrhea OHT	HF 1st degree AV block	Deceased
*4 (C*_2_*)	49	2017	24	Paresthesia Weight loss	SM-PN	OHT Constipation	HF 1st degree AV block SVES RBBB pericardial effusion	Alive
*5 [D]*	51	2016	48	Diarrhea Weight loss	SM-PN	Diarrhea OHT	HF AF	Alive
*6 [E*_1_*]	52	2017	9	Paresthesia Weight loss	SM-PN CTS	Constipation OHT	HF RBBB	Alive
*7 [E*_2_*]	47	2017	24	Paresthesia Weight loss	SM-PN	Constipation OHT	–	Alive
*8[E*_3_**]	47	2017	7	Paresthesia	SM-PN CTS	–	SVES	Alive
*9 [F]*	49	2017	36	Paresthesia	SM-PN CTS	–	HF 1st degree AV block AF	Alive
*10 [G]*	49	2017	60	Paresthesia	SM-PN CTS	Constipation	HF Trifascicular block Pericardial effusion	Alive
*11 [H]*	42	2018	6	Weight loss Gastroparesis	S-PN CTS	OHT Gastroparesis Diarrhea/constipation	HF 3rd degree AV block NSVT RBBB	Alive
*12 [I*_1_*]	41	2017	48	Paresthesia CTS Weight loss	SM-PN	Constipation OHT Urinary dysfunction	HF RBBB	Alive
*13 [I*_2_*]	46	2017	60	Paresthesia Weight loss	SM-PN	OHT	HF	Alive

*AF* atrial fibrillation, *CTS* carpal tunnel syndrome, *HF* heart failure, *NSVT* non-sustained ventricular tachycardia, *OHT* orthostatic hypotension, *RBBB* right bundle branch block, *S-PN* sensory polyneuropathy, *SM-PN* sensory motor polyneuropathy, *SVES* supraventricular

Letters A–I denote each family

*Siblings

**First cousins

The median age at death was 53 years (*n* = 3; range: 46–54 years); median survival time from symptom onset was 3 years (range: 3–5 years). In all three patients, death was caused by complications related to cardiac involvement.

Overall, 38 relatives of the ATTR patients in our study had died by sudden death and/or paralysis, with median age at death being 48 years. Median age at death among males was 5 years lower than in females (males: 45 years, females: 50 years).

The median time from symptom onset to diagnosis (index patients; *n* = 10) was 24 months (range: 6–60 months). In women, median time to diagnosis was 20 months; this was longer in men, at 36 months. In all symptomatic patients (living or deceased), the median age at onset of ATTR was 45 years, and the median age at diagnosis was 48 years (49 years in women and 47.5 years in men). Symptoms of ATTR at first presentation, as shown in Table 1, were distal paresthesia (*n* = 10), carpal tunnel syndrome (*n* = 3), orthostatic hypotension, (*n* = 9), heart failure (*n* = 11), weight loss (*n* = 8), diarrhea (*n* = 4) and constipation (*n* = 5). Overall, clinical progression was similar in all patients, with a prolonged history of neurological disease and later signs of cardiac disease.

**Fig. 2:**
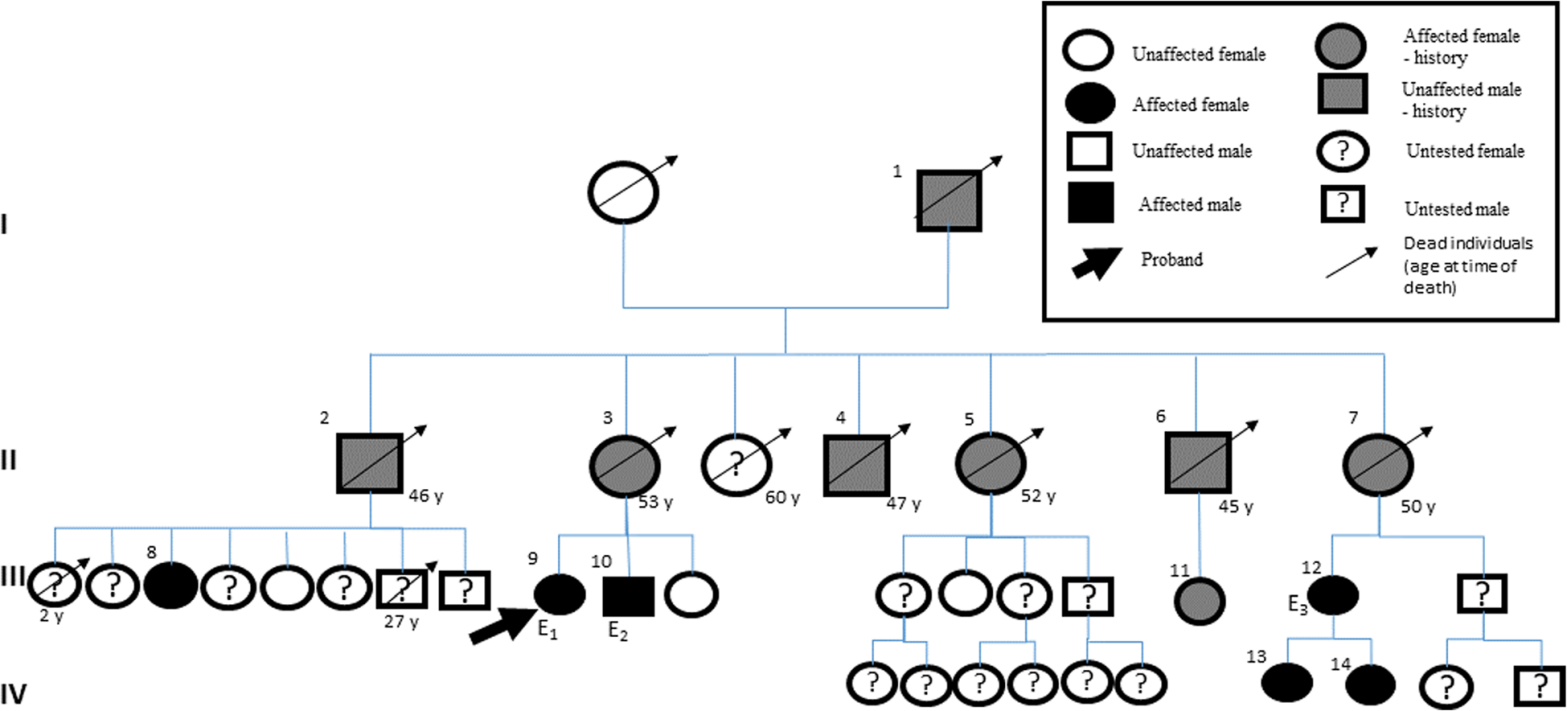
Pedigree for family E, showing which family members bear the *Glu54Gln* ATTR variant. Arabic numerals refer to the individuals affected by *Glu54Gln* and Roman numerals refer to successive generations. Clinical data on patients E_1_ (8), E_2_ (9) and E_3_ (10) are included in Table 1. Patient 11 has been paralyzed for some time but refuses medical care and genetic testing. Family members denoted by numbers 8, 13 and 14 are healthy carriers

Figure 2 shows the pedigree chart of the largest family with *Glu54Gln*-mutated ATTR. In the second generation, six out of seven members died between the ages of 45 and 53, with clinical signs indicating ATTRh. Unfortunately, in the third and fourth generation we do not have complete information about many family members, either because they have not provided consent for medical testing and treatment, or because they have moved house or emigrated.

### Cardiac involvement

Cardiac involvement (Table 2) was assessed in all 13 symptomatic patients, and all of these patients exhibited cardiac changes at diagnosis. The average concentration of N-terminal-pro B-type natriuretic peptide (NT-proBNP) in seven symptomatic patients was 2815.6 pg/ml (range: 43.5–7140 pg/ml). Troponin was positive in three out of five tested patients (60%).

**Table 2:** Cardiac characteristics of *Glu54Gln*-mutated ATTR

Cardiac characteristics	Glu54Gln ATTR (7 patients)
Age, years	45.9
Sex, % male	28.5%
NT-proBNP (pg/ml), mean [range]	2815.6 [34.5–7140]
IVS thickness, mm, mean [range]	16 [13–21]
PW thickness, mm, mean [range]	15 [12–17]
RV free wall thickness, mm, mean [range]	7 [5–8]
S′, cm/s, mean [range]	5.4 [4–7]
E/A, mean [range]	2.3 [0.9–4.2]
E/E’, mean [range]	19 [10–26]
TAPSE, mean [range]	19 [13–26]
GLS, %, mean [range]	−13 [−7.7, −18.5]
Atrial fibrillation, n, %	2 (15%)
AV block, n, %	5 (38%)
RBBB, n, %	4 (31%)
LBBB, n, %	1 (8%)
LVEF %, mean [range]	49 [30–60]
Pericardial effusion, n, %	2 (15%)

*A* late phase mitral wave velocity, *AV block* atrioventricular block, *E* early mitral wave velocity, *E’* early diastolic mitral annulus velocity, *GLS* global longitudinal strain, *IVS* interventricular septum, *LBBB* left bundle branch block, *LVEF* left ventricular ejection fraction, *NT-proBNP* N-terminal-pro hormone B-type natriuretic peptide, *PW* posterior wall, *RV thickness* right ventricular thickness, *RBBB* right bundle branch block, *S′* mitral annulus systolic myocardial velocity, *TAPSE* Tricuspid annular plane systolic excursion

Based on electrocardiogram (ECG) and Holter 24-h monitoring, 9/13 patients (69%) presented with sinus rhythm, two (15%) with atrial fibrillation, and six (46%) with ventricular extrasystoles. Atrioventricular block (AV block) was present in five patients (38%): of these, four suffered a first-degree AV block and one had a third-degree AV block. Four patients (31%) had a right bundle branch block (RBBB) and one patient (8%) presented with a left bundle branch block; one patient had a trifascicular block (RBBB, first-degree AV block and left anterior hemiblock) (Table 2).

One patient needed an implantable cardioverter-defibrillator at diagnosis owing to repeated runs of non-sustained symptomatic ventricular tachycardia and third-degree AV block. Another patient required a pacemaker implant for trifascicular block with long conduction times that were identified in the electrophysiological examinations.

All symptomatic patients underwent echocardiographic evaluation. Mean interventricular septum (IVS) thickness was 16 mm and mean posterior wall (PW) thickness was 15 mm. Mean left ventricular (LV) ejection fraction (LVEF) was 49%, mean mitral annulus systolic myocardial velocity was 5.4 cm/s, mean early mitral wave velocity/late phase mitral wave velocity was 2.3, mean early mitral wave velocity/early diastolic mitral annulus velocity was 19, and mean left ventricular global longitudinal strain (GLS) was − 13%. Mean right ventricular free wall thickness was 7 mm, with an average Tricuspid Annular Plane Systolic Excursion was 19 mm. Pericardial effusion was present in two patients (Table 2).

Scintigraphy with 99 m technetium-hydroxymethylene diphosphonate (99mTc-HDP) was performed in eight patients. We analyzed the visual score (VS) and heart/contralateral region (H/CL) in seven symptomatic patients: average H/CL was 2.25 [range: 1.69–3.19]. Based on Perugini classification, one patient had a VS of III, three patients had a VS of II and four patients had a VS of I.

One patient underwent cardiac magnetic resonance imaging, which showed asymmetric cardiac hypertrophy, mild-to-moderate biventricular dysfunction and diffuse circumferential subendocardial late gadolinium enhancement.

### Neurologic involvement

Among 13 symptomatic patients (living and deceased) analyzed at diagnosis, 12 (92%) had axonal polyneuropathy and seven patients (53%) had carpal tunnel syndrome. As shown in Table 3, in the 10 living symptomatic patients, mean modified body mass index (mBMI) was 1084 (g/l) x (kg/m^2^) and mean neuropathy impairment score of lower limbs (NIS-LL) was 13.4 at diagnosis and 15.6 six months after diagnosis. At diagnosis, all 13 symptomatic patients had stage 1 familial amyloid polyneuropathy. Polyneuropathy disability (PND) score was available for eight symptomatic patients: five had a score of I and three had a score of II.

**Table 3:** Neurological characteristics of *Glu54Gln*-mutated ATTR

Neurological characteristics at diagnosis for symptomatic patients		Glu54Gln ATTR
NIS-LL, mean, range (*N* = 8)		13.4 [4–26]
mBMI, (g/l) x (kg/m^2^), mean, range (*N* = 8)		1084 [611–1444]
PND scale (8 patients)	Grade I, n (%)	5 (63%)
	Grade II, n (%)	3 (37%)
Polyneuropathy, *n* (%) (*N* = 13)		12 (92%)
Carpal tunnel syndrome, *n* (%) (*N* = 13)		7 (53%)
Orthostatic hypotension, *n* (%) (*N* = 13)		9 (69%)
Diarrhea, n (%) (*N* = 13)		4 (31%)
Constipation, n (%) (*N* = 13)		5 (38%)
Weight loss, n (%) (*N* = 13)		8 (62%)

*mBMI* modified body mass index, *NIS-LL* neuropathy impairment score of lower limbs, *PND* polyneuropathy disability score

Orthostatic hypotension was present in 9/13 symptomatic patients (69%). At diagnosis, four patients (31%) had diarrhea and five (38%) had constipation. Eight patients (62%) presented with weight loss before diagnosis. One patient (8%) presented with frequent vomiting episodes/gastroparesis.

### Other characteristics

Sicca syndrome was present in 3/13 symptomatic patients (23%). One patient (7.7%) exhibited Howell-Jolly bodies, without any associated pancytosis or splenomegaly. Mean factor X level was 84% [range: 50–131%]. None of the patients presented with nephrotic syndrome or liver involvement. A fibroscan examination was performed in seven patients and the mean value was 5.1 kPa, i.e. within the normal range. No cholestasis or hepatomegaly was observed.

## Discussion

This paper is the first to describe the clinical characteristics of the largest *Glu54Gln*-mutated ATTRh cohort diagnosed to date, and to our knowledge is the first reported incidence of this variant worldwide.

### Prevalence

Until recently, ATTRh — also referred to as family polyneuropathy — was considered a genetic disease that only occurred in specific populations in Western Europe, Japan, the USA, and South America. ATTR was largely considered to be a neurological disease that held little interest for other medical specialists. In addition, it is very rare and has a heterogeneous clinical manifestation. Its onset later in life, in patients over 30 years old, makes it less likely that doctors consider a differential diagnosis of a genetic disease. All of these factors have contributed to the lack of ATTR diagnoses in Romania and elsewhere. In 2005, we diagnosed our first case of ATTRh in Romania and only a few years later, a second case was diagnosed.

The prevalence of ATTR is lower in Romania (1.02 per 1,000,000 people) compared with Portugal (192–204 per 1,000,000), Sweden (25.8 per 1,000,000), Cyprus (37.2–43.3 per 1,000,000), Italy (9.1 per 1,000,000), France (7.5 per 1,000,000), Bulgaria (5.68 per 1,000,000), Netherlands (2.67 per 1,000,000) and Germany (1.48 per 1,000,000), but it is higher than in Japan (0.99 per 1,000,000) and Turkey (0.32 per 1,000,000) [6].

In Suceava county, all patients with ATTR were positive for the *Glu54Gln* mutation, with a prevalence of 2.39 per 100,000 people. This prevalence is lower than that in endemic areas of Europe (Northern Portugal, 151; Northern Sweden, 104; Cyprus, 3.72 per 100,000) [7–9], and lower than in Spain (Majorca, 3.1–5 per 100,000), but higher than in Japan (Nagano, 1.29; Kumamoto, 1.02; Ishikawa, 0.38 per 100,000) and Italy (Sicily, 0.02–0.88 per 100,000) [6].

We believe that the prevalence of this disease in Romania is substantially underestimated, owing to several contributing factors including lack of access to or refusal to receive medical care for paralysis patients; diagnostic failure; and early sudden death. Another contributing factor is the limited survival of these patients after disease becomes symptomatic (average survival time from diagnosis to death in this study was 3.7 years).

### Clinical phenotype of Glu54Gln-mutated ATTR

The phenotype of ATTRh may be dominated by neurological involvement or cardiac involvement, or may be mixed, with both cardiac and neurological involvement [10]. *Glu54Gln*-mutated ATTRh has a mixed phenotype, with clinical onset in patients’ fourth decade. Distal paresthesia and carpal tunnel syndrome are initial manifestations, with significant cardiac involvement and autonomic dysfunction occurring after diagnosis.

### Cardiac characteristics of Glu54Gln-mutated hATTR

Cardiac involvement has a significant role in the morbidity and mortality of patients with amyloidosis. Compared with published literature [11], *Glu54Gln*-mutated ATTRh has similar echocardiographic features to other forms of ATTRh.

99mTc-HDP scintigraphy can be used to detect cardiac involvement in ATTRh even before clear echocardiographic changes are apparent [12]. In this study, all symptomatic patients exhibited myocardial uptake of 99mTc-HDP. Scintigraphy was only performed in one asymptomatic patient and this showed no uptake.

Compared with *Val122Ile*-mutated ATTRh, which is associated with a predominantly cardiac phenotype, patients with the *Glu54Gln* mutation in our cohort were younger at diagnosis, with lower LV wall thickness and LVEF. Average LV wall thickness in our cohort was similar to that reported in other ATTRh cohorts, but lower than in patients with wild-type ATTR and higher than in patients with light chain amyloidosis [13]. Atrial fibrillation appeared to be less frequent in our study than in reports of patients with the *Val122Ile* mutation [9]. Patients with the *Glu54Gln* mutation had higher NT-proBNP levels at diagnosis than patients with other forms of ATTRh, although they were still lower than in patients with wild-type ATTR or light chain amyloidosis.

### Neurologic characteristics of ATTR Glu54Gln

Neurological involvement in this cohort was manifested by peripheral polyneuropathy in a large proportion of patients, mostly with a grade I PND score and grade I familial amyloid polyneuropathy score. Carpal tunnel syndrome was present in half of the patients. Autonomic nervous system involvement was present in more than half of the symptomatic patients, with its main manifestations being orthostatic hypotension and diarrhea. Weight loss at diagnosis was reported in 62% of symptomatic patients.

### General findings: comparison with other ATTRh variants

In our study, age at diagnosis was lower than the European average, based on the NEURO-TTR study of 172 patients with ATTR [14].

Patients with ATTRh and the *Glu54Gln* mutation present with a mixed phenotype, similar to patients with *Val30Met* variant-late onset, *Glu89Gln*, *Phe64Leu* or *Thr49Ala* [8]. Patients with the *Val30Met* variant-late onset typically exhibit a later onset at ~ 59.5 years, and often have no family history, less autonomic involvement and more cardiac, renal and ocular involvement [15]. The *Glu89Gln* variant is characterized by onset at 49 years; symptoms at onset typically include paresthesia/carpal tunnel syndrome, early cardiac dysfunction followed by heart failure, dysautonomia and cachexia. Average survival is 7.6 years, and the main cause of death is cardiac involvement [16]. Another example is the *Phe64Leu* variant: this has variable penetrance, with a positive family history in only half of cases. Average age at onset is 64 years, with symptoms of paresthesia/carpal tunnel syndrome, severe neuropathy and moderate dysautonomia. Cardiac involvement is mild. Average survival is 11.3 years, with the average age at death being 77.6 years, typically caused by cachexia and dysautonomia [16]. Finally, ATTRh patients with the *Thr49Ala* variant experience onset of the disease at ~ 55 years. Autonomic involvement is typical, but this remains isolated for several years. Patients also experience moderate neuropathy, cachexia, and mild cardiac involvement. Average survival is 10.7 years, with a common cause of death being dysautonomia and cachexia [16]. Compared with these mixed-phenotype variants, patients with the *Glu54Gln* mutation have earlier disease onset (the average is at 44.5 years old); they often present with paresthesia/carpal tunnel syndrome, but with simultaneous cardiac involvement (all *Glu54Gln* patients have some degree of cardiac involvement at diagnosis).

To date, we have found only one published case report on ATTRh patients with the *Glu54Gln* variant. Although the case describes a 43-year-old female patient in Spain, she is in fact of Romanian origin, who presented with severe cardiomyopathy and carpal tunnel syndrome and died immediately after diagnosis [17].

Novel treatments for ATTRh that have recently been approved are the TTR tetramer stabilizer tafamidis, and patisiran and inotersen, which work by RNAi-mediated silencing of hepatic TTR expression. Tafamidis has been shown to stabilize the variant transthyretin tetramer, including the *Glu54Gln* variant, in an ex vivo study [18]. Since 2016, tafamidis has been available in Romania. This progress is an opportunity to improve care for patients suffering from this fatal disease, but the efficacy of therapy largely depends on early diagnosis. For this reason, it is imperative to raise doctors’ awareness, regardless of their specialism, about this rare disease, especially in non-endemic areas. Management of healthy carriers is also a key issue. According to current recommendations, treatment initiation should only begin when patients present with stage 1 polyneuropathy. This leaves us with the question of how to manage carriers who exhibit neurological symptoms, but do not yet fulfil these criteria.

The main limitation of this study is the relatively low number of patients in our cohort. However, this represents all diagnosed ATTRh patients with the *Glu54Gln* mutation diagnosed in Romania, highlighting the rarity of this disease. Patients were diagnosed and treated over a prolonged period of 14 years, during which the standard of care changed. This means that some diagnostic tests, such as cardiac biomarkers, NIS-LL and PND scores, were not carried out in all patients. Nonetheless, a thorough workup was conducted in line with the standard of care at the time for each patient, including family history, laboratory tests, and cardiac, neurologic, and autonomic evaluation.

## Conclusions

This is the largest study published to date about ATTRh in Romania. This Romanian cohort predominantly included patients with the *Glu54Gln* mutation, which appears to be prevalent in the Romanian population, with all the patients originating from the North-East of Romania. Overall, the prevalence of ATTR in our country is low compared with endemic areas. Clinical manifestations of ATTRh in this cohort were dominated by early onset, significant neurologic and cardiac involvement, aggressive disease progression and short survival times after diagnosis. Earlier diagnosis and therapeutic intervention with new agents has potential to improve prognosis for patients such as these.

## Methods

This retrospective, observational study included all patients carrying a *Glu54Gln* TTR mutation who were diagnosed and evaluated in the Haematology Department of Fundeni Clinical Institute and in the Neurology Department of the University Emergency Hospital in Bucharest, Romania, between 2005 and 2018. The study was approved by the Ethics Committee of Fundeni Clinical Institute and patients’ informed consent was obtained.

Data were collected as follows: family history, place of residence and birthplace of relatives with ATTR, symptoms, complete blood count, the presence of Howell-Jolly bodies in peripheral blood smears, and the level of coagulation factor X.

The TTR *Glu54Gln* mutation was evaluated through polymerase chain reaction- restriction fragment length polymorphism using restriction enzyme Pvu II [4]. Also, DNA sequencing (Sanger method) of all four exons of the TTR gene was performed [19]. Abdominal fat pad biopsy or salivary gland biopsy was performed using Congo Red staining and polarized light microscopy to identify amyloid deposits, as well as immunohistochemistry with anti-TTR antibodies, serum protein electrophoresis, serum protein immunofixation, and serum free light chain analysis.

Cardiac evaluation varied with the clinical indication of the patient. It included, variously: assessment of cardiac biomarkers (NT-proBNP, troponin); ECG; 24-h Holter monitoring; 99mTc-HDP scintigraphy; and cardiac magnetic resonance imaging. Echocardiography was used to evaluate functional and structural parameters including the IVS, PW thickness, left ventricular ejection fraction, LV diastolic function parameters and the GLS.

Liver function was evaluated using laboratory tests (for alanine aminotransferase; aspartate aminotransferase; bilirubin [total and direct]; gamma-glutamyl transferase; alkaline phosphatase; and albumin) and liver stiffness was assessed using a fibroscan. Renal function was evaluated by estimating glomerular filtration rate and 24-h proteinuria.

Investigations into the function of the peripheral nervous system included electromyography, NIS-LL score, polyneuropathy disability score, and mBMI. Evaluation of the autonomic nervous system included presence of orthostatic hypotension and/or symptoms of intestinal dysfunction. Lacrimal gland secretion was determined using Schirmer’s test.

## Data Availability

The datasets used and/or analysed during the current study are available from the corresponding author on reasonable request.
